# Volatile Organic Compound Profile Changes in *Lentinula edodes* Pileus Following *Trichoderma atroviride* Inoculation

**DOI:** 10.3390/jof12080583

**Published:** 2026-08-07

**Authors:** Dong-Ryeol Yu, Kyung-Gu Min, Tae-Min Park, Youn-Jin Park, Myoung-Jun Jang

**Affiliations:** Department of Plant Resources, Kongju National University, Yesan 32439, Republic of Korea

**Keywords:** volatilome, green mold disease, infection-associated

## Abstract

This study explored disease symptom development and changes in relative volatile organic compound (VOC) profiles after inoculation of *Trichoderma atroviride* onto *Lentinula edodes* pilei. Phylogenetic analyses based on ITS and *tef1* sequences supported identification of the inoculated strain as *T. atroviride*. Inoculation caused surface depression and browning, while discoloration extended from the surface into internal tissues. At 3 days after inoculation, the inoculated strain was re-isolated from T1–T3 tissues, confirming the presence of viable fungi at all sampling positions. Relative VOC profiles were compared among untreated pilei, wound-treated samples, *T. atroviride* cultures (TA), and samples collected from different positions after inoculation (T1–T3). PCA and heatmap analyses revealed descriptive VOC variation among treatment groups and T1–T3, although no individual VOCs differed significantly among T1–T3 after FDR correction. 6-Pentyl-2H-pyran-2-one showed the highest relative peak area percentage in TA, whereas 2(3H)-naphthalenone was consistently detected only in TA. In inoculated pilei, ylangene was detected at all positions, whereas γ-muurolene, α-muurolene, 1-naphthalenol, 3-octanone, and 1,2,4-trithiolane showed different detection patterns and relative peak area percentages among T1–T3. These findings provide a basis for quantitative validation of inoculation-associated VOC changes and potential spatial variation within the pileus.

## 1. Introduction

*Lentinula edodes* (*L. edodes*) is an edible mushroom produced through both traditional log cultivation and cultivation on sawdust-based artificial substrates, and in recent years, sawdust-based cultivation has been increasingly adopted in commercial production [1,2]. However, both cultivation systems may be affected by contaminating fungi present in the cultivation environment, which can cause visible disease symptoms and deterioration of fruiting-body quality [3].

In particular, green mold disease caused by *Trichoderma* spp. is recognized as a major disease affecting edible mushroom production, and delayed detection and control may facilitate the spread of contamination within cultivation systems [4]. Recent studies have likewise identified *Trichoderma* contamination as a limiting factor in shiitake production, reporting that various *Trichoderma* spp. were detected in contaminated shiitake substrates and that shiitake strains exhibited susceptibility to the major *Trichoderma* spp. [5].

In mushrooms, responses to pathogen infection may manifest not only as morphological alterations but also as changes at the physiological and metabolic levels, including alterations in enzyme activity and in the expression of defense-related genes [6,7]. Volatile organic compounds (VOCs) function as signaling molecules and metabolites in fungal ecological interactions, and fungi such as *Trichoderma* produce their own characteristic VOC blends [8,9]. In addition, in mushrooms, healthy and infected states can be distinguished on the basis of VOC profiles, indicating that these profiles may serve as indicators reflecting the pre-infection metabolic state and the local response at the site of infection [10].

VOC profiles may vary depending on medium composition and culture conditions [9], and tissue-specific variation in volatile compounds has also been reported within the fruiting body of *L. edodes* [11]. Moreover, because VOC profiles may differ according to medium components, the pathogen grown in pure culture, the fruiting body itself, and infected tissues in which disease symptoms have developed, distinguishing and interpreting these sources separately is important for discriminating infection-associated VOC changes from background signals.

Previous studies on VOCs in *L. edodes* have focused primarily on the analysis of volatile characteristics related to quality, aroma, tissue-specific differences, and processing and storage conditions, whereas VOC research on Trichoderma has largely been directed toward elucidating the composition and biological activities of its self-produced metabolites [12,13,14]. However, comparative studies examining changes in VOC profiles associated with Trichoderma infection in *L. edodes* remain relatively limited. Accordingly, in the present study, SPME-GC-MS was employed to comparatively analyze VOC profiles in *Trichoderma atroviride* (*T. atroviride*) cultures, healthy and mechanically wounded *L. edodes* pilei, and samples collected from different positions of the *L. edodes* pileus following inoculation with *T. atroviride*, with the aim of characterizing infection-associated changes and spatial variation in relative VOC profiles.

## 2. Materials and Methods

### 2.1. Pathogen Strain and Molecular Identification Based on ITS and tef1 Sequences

The pathogen used in this study was a *T. atroviride* stock preserved at Kongju National University, and molecular identification of the strain was performed based on ITS and translation elongation factor 1-alpha (*tef1*) sequence analyses. The strain was inoculated onto PDA (potato dextrose agar; Difco, Detroit, MI, USA) medium and cultured for 3 days at 25 °C under dark conditions, after which genomic DNA was extracted using the DNeasy Plant Mini Kit (Qiagen, Hilden, Germany). The ITS region was amplified using the ITS1/ITS4 primers [15], whereas the *tef1* region was amplified using the EF1-983F/EF1-2218R primers [16]. PCR amplification of both regions was carried out with an initial denaturation at 94 °C for 4 min, followed by 35 cycles of 94 °C for 30 s, 51 °C for 30 s, and 72 °C for 1 min, and a final extension at 72 °C for 10 min. The PCR products were submitted to SolGent Co., Ltd. (Daejeon, Republic of Korea) for purification and sequencing. The obtained ITS and tef1 sequences were separately aligned with the corresponding reference sequences retrieved from GenBank using MAFFT v7 [17], and a phylogenetic tree was constructed in MEGA X [18] using the neighbor-joining method. Bootstrap analysis was performed with 1000 replicates.

### 2.2. Lentinula edodes Cultivation

The *L. edodes* used in this study was the ‘Sanjo 701ho’ strain, which was obtained from Cheongheung Mushroom Agricultural Cooperative located in Cheongyang-gun, Chungcheongnam-do, Korea. The spawn was inoculated into a substrate composed of oak sawdust and rice bran mixed at an 80:20 (*v*/*v*) ratio, and fruiting bodies were induced after 60 days of incubation in the dark at 20 ± 1 °C, followed by 40 days of incubation under light conditions.

### 2.3. Trichoderma atroviride Inoculation

Agar plugs containing *T. atroviride* mycelia grown on PDA were used as the inoculum. Using a sterile cork borer, 6 mm diameter agar plugs were excised from the actively growing colony margin and placed with the mycelial side in direct contact with the unwounded surface of the *L. edodes* pileus. Three distinct inoculation sites were established near the margin of each pileus, and one agar plug was placed at each site. For the wound-only treatment, three approximately 6 mm long superficial scratch wounds were made near the margin of each pileus using a sterile scalpel. The wound sites were positioned to correspond approximately to the inoculation sites used in the *T. atroviride* inoculated group. Three independent pilei were used for each treatment group, with each pileus considered a biological replicate. The inoculated and wounded pilei were placed separately in lidded containers measuring approximately 22 × 10 cm and incubated at 16 °C under dark conditions for 3 days.

### 2.4. Re-Isolation and Viable Count of T. atroviride

To verify the presence of viable *T. atroviride* in the T1, T2, and T3 tissues at 3 days after inoculation, the fungus was re-isolated, and viable counts were determined. Tissue samples (0.1 g) collected at each sampling position were homogenized in 900 μL of sterile distilled water and serially diluted. Aliquots (100 μL) of each dilution were spread onto *Trichoderma harzianum* Selective Agar Base supplemented with *Trichoderma* Selective Supplement according to the manufacturer’s instructions (HiMedia Laboratories, Mumbai, India) and incubated at 25 °C for 48 h. Colonies exhibiting morphological characteristics consistent with the *T. atroviride* strain used for inoculation were counted, and viable counts were expressed as CFU per gram of tissue based on colony number, dilution factor, plated volume, and tissue weight. Three independent pilei were used as biological replicates for each sampling position (*n* = 3).

### 2.5. Symptom Assessment and Stereomicroscopic Observation

After inoculation with *T. atroviride*, browning symptoms on the surface of the *L. edodes* pileus were observed visually, and pilei showing browning were cut with a scalpel to examine the pattern of discoloration in the internal tissue using a stereomicroscope at 6.7× magnification (SZ61TR, Olympus, Tokyo, Japan).

### 2.6. Spatial Sample Collection for VOC Analysis

For VOC analysis, the three inoculation sites established near the margin of each pileus were designated as T1 sites. For each T1 site, T2 and T3 were positioned 2 and 4 cm away, respectively, along a straight transect extending toward the opposite pileus margin. The three sampling transects were arranged approximately parallel to one another. The pileus diameters of the three fruiting bodies used as biological replicates were 5.55, 5.56, and 5.52 cm, respectively, with a mean diameter of 5.54 ± 0.02 cm. Based on similar pileus diameters, the sampling sites were established at equal 2 cm intervals to compare VOC profiles among the inoculation sites, adjacent tissues, and tissues located farther from the inoculation sites across the pileus. The sampling positions and distances were predetermined before visual assessment of lesion development. Three days after inoculation, tissues collected from the three T1 sites within each pileus were pooled to constitute one composite T1 sample. Similarly, tissues collected from the three corresponding T2 sites and the three corresponding T3 sites were pooled separately to constitute one composite T2 sample and one composite T3 sample, respectively. For the wound-only group, tissues collected from the three wounded sites within each pileus were pooled to constitute one composite sample. For the untreated control, tissues collected from three positions corresponding to the wound-treatment sites within each pileus were similarly pooled to constitute one composite sample. For each pileus-derived group, 0.2 g of the composite sample was placed in a 20 mL headspace vial and subjected to SPME-GC-MS analysis. Each composite sample prepared from one pileus was considered one biological replicate, and three independent pilei were used for each group (*n* = 3). In addition, PDA medium and *T. atroviride* cultures (TA) were analyzed as background and pathogen reference samples, respectively, while healthy and mechanically wounded *L. edodes* pileus tissues were included as untreated and wound-only comparison groups, respectively.

### 2.7. SPME-GC-MS Analysis of VOCs

The VOCs of the collected samples were analyzed using SPME-GC-MS. Peak processing was performed using LabSolutions software(Ver.6.4) (Shimadzu Corporation, Kyoto, Japan), and mass spectral library searches were conducted using the NIST20s, NIST20-1, and NIST20-2 libraries. For each detected peak, library search results were compared based on the similarity index (SI), and the top-hit candidate with the highest SI was adopted as the tentative compound annotation. No predefined minimum SI cutoff was applied. The relative abundance of each VOC was compared using the peak area percentage (%). Detailed conditions for the SPME-GC-MS analysis are presented in Table 1.

### 2.8. Data Preprocessing and Multivariate Analysis of VOC Profile

The VOC data obtained from the SPME-GC-MS analysis were organized based on the peak area percentage (%) of the compounds detected in each sample. Within each sample, duplicate detections of the same compound were combined, and undetected compounds were treated as zero. Multivariate analysis of VOC profiles included *T. atroviride* cultures (TA), wound-only samples (Wound), healthy *L. edodes* pilei (Control), and position-specific samples collected after inoculation (T1, T2, and T3), whereas empty-vial and PDA samples were used for background peak filtering. All 65 VOCs remaining after background filtering were used for PCA, and the data were log1p-transformed and autoscaled prior to analysis. For heatmap visualization, the 25 VOCs showing the highest variance in raw peak area percentage across all Control, Wound, TA, T1, T2, and T3 samples were selected, and the values for each VOC were z-score normalized across all samples. Data preprocessing and multivariate analyses were performed using R version 4.5.1. PCA was conducted using the prcomp function. VOC rows in the heatmap were hierarchically clustered using Euclidean distance and complete linkage and visualized using the pheatmap package. Sample columns were not clustered and were arranged in the order Control, Wound, TA, T1, T2, and T3.

### 2.9. Statistical Analysis

Because T1, T2, and T3 were samples collected from different positions on the same pileus, positional differences were evaluated exploratorily using the Friedman test [19], implemented using the friedman.test function in the R stats package. To account for multiple testing across VOCs, the resulting *p*-values were adjusted using the Benjamini–Hochberg false discovery rate procedure [20] with the p.adjust function (method = “BH”) in the R stats package.

## 3. Results

### 3.1. Identification of the Pathogen as T. atroviride

**Figure 1:**
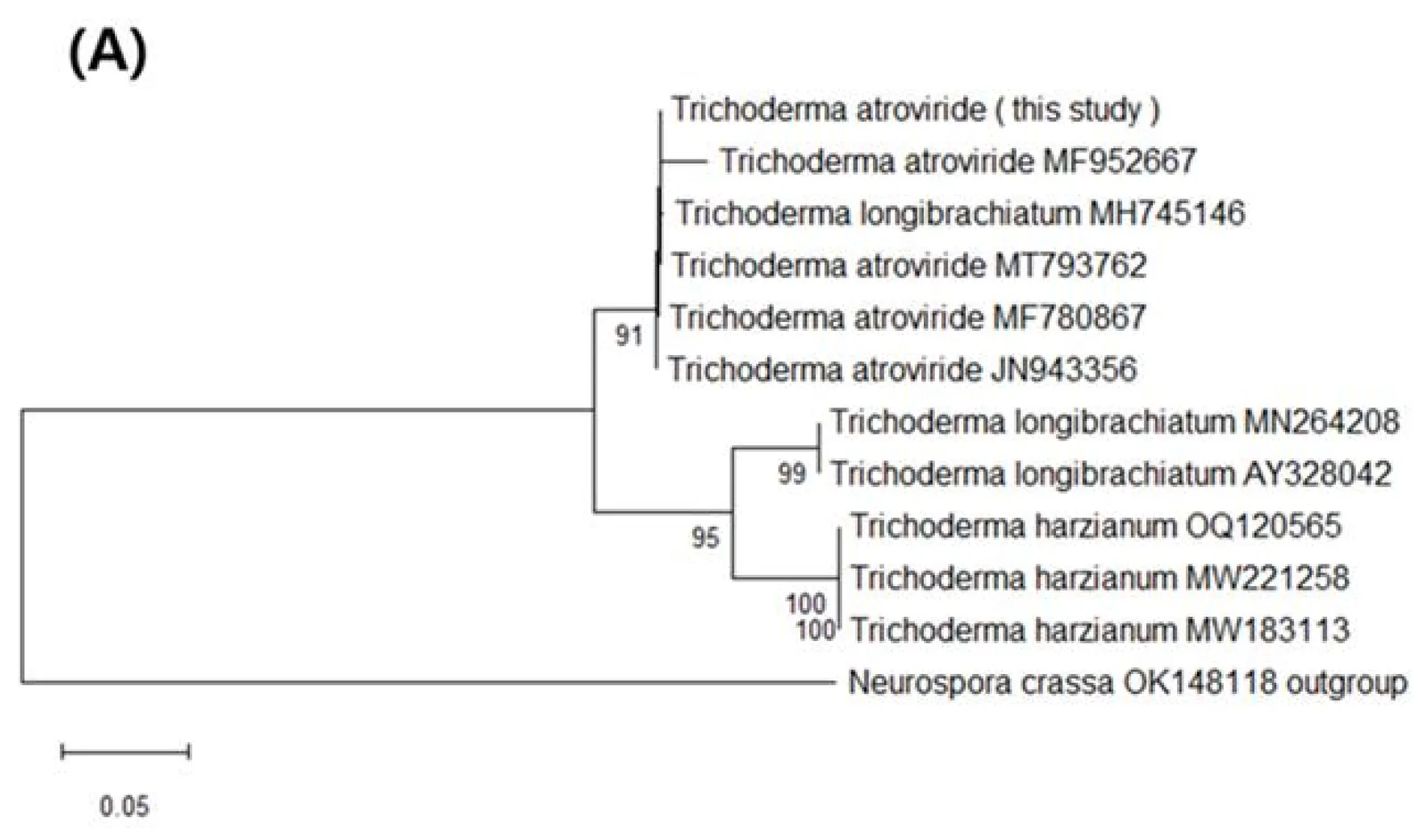

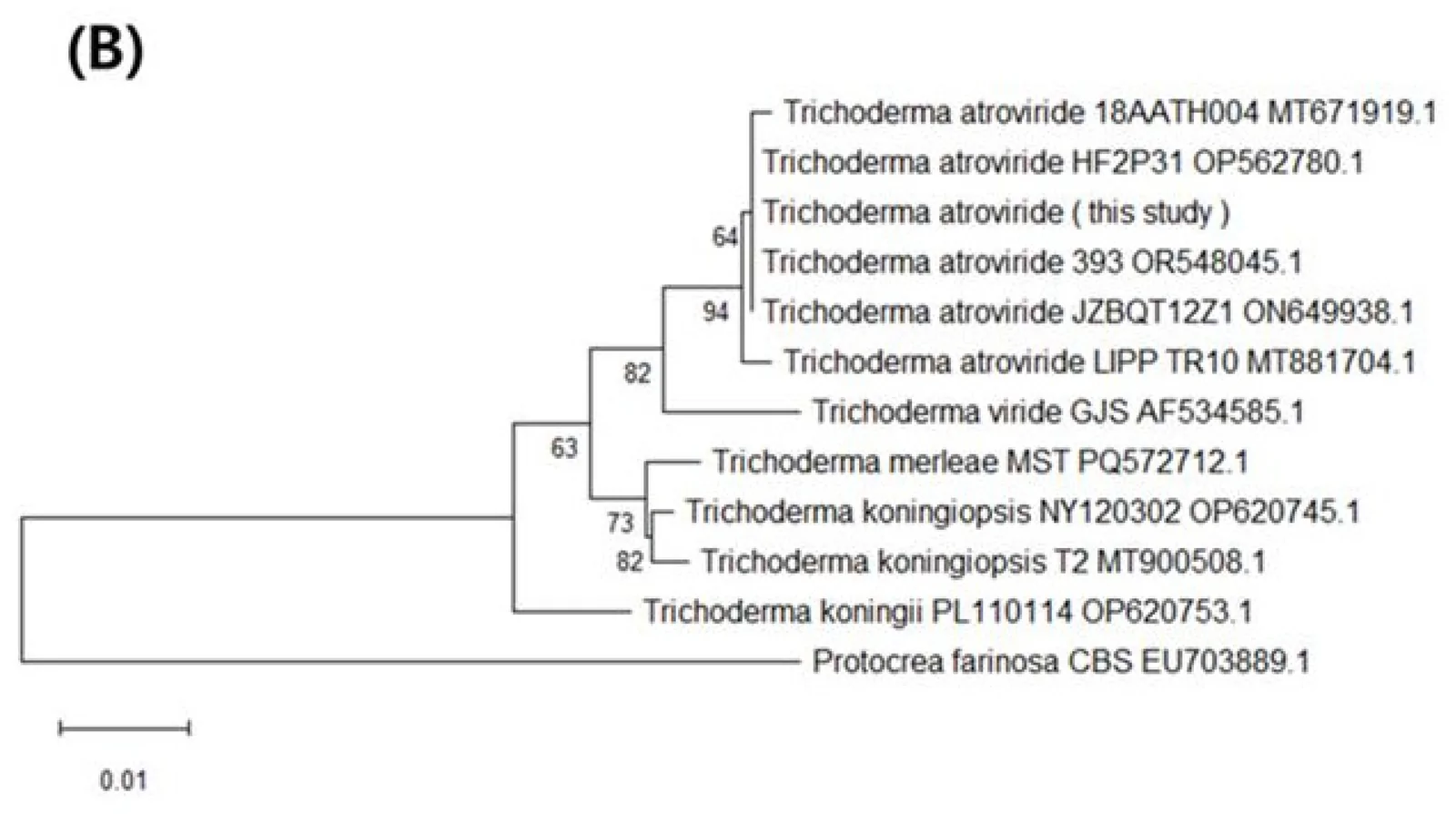
Identification of the pathogen as *Trichoderma atroviride* based on ITS and tef1- α phylogenetic analyses. Neighbor-joining phylogenetic trees were constructed using (**A**) ITS and (**B**) tef1- α sequences of the isolate used in this study and reference *Trichoderma* isolates retrieved from GenBank. *Neurospora crassa* OK148118 and *Protocrea farinosa* were used as outgroups for the ITS and tef1- α analyses, respectively. Bootstrap values ≥ 50% based on 1000 replicates are shown at the nodes. The scale bars indicate 0.05 and 0.01 substitutions per site for panels (**A**) and (**B**), respectively. The isolate used in this study is indicated as “*Trichoderma atroviride* (this study)”.

To identify the pathogen used in this study at the species level, phylogenetic analyses were performed by comparing the ITS and tef1- α sequences with reference sequences deposited in NCBI GenBank. In the ITS-based phylogenetic analysis, the isolate used in this study clustered with the *T. atroviride* reference isolates and was separated from *T. harzianum* and *T. longibrachiatum* (Figure 1A). Similarly, the tef1- α-based phylogenetic analysis placed the isolate within the *T. atroviride* clade (Figure 1B). Therefore, the phylogenetic analyses based on both molecular markers supported the identification of the pathogen used in this study as *T. atroviride*.

### 3.2. Disease Symptom Development on the Pileus of L. edodes After T. atroviride Inoculation

**Figure 2:**
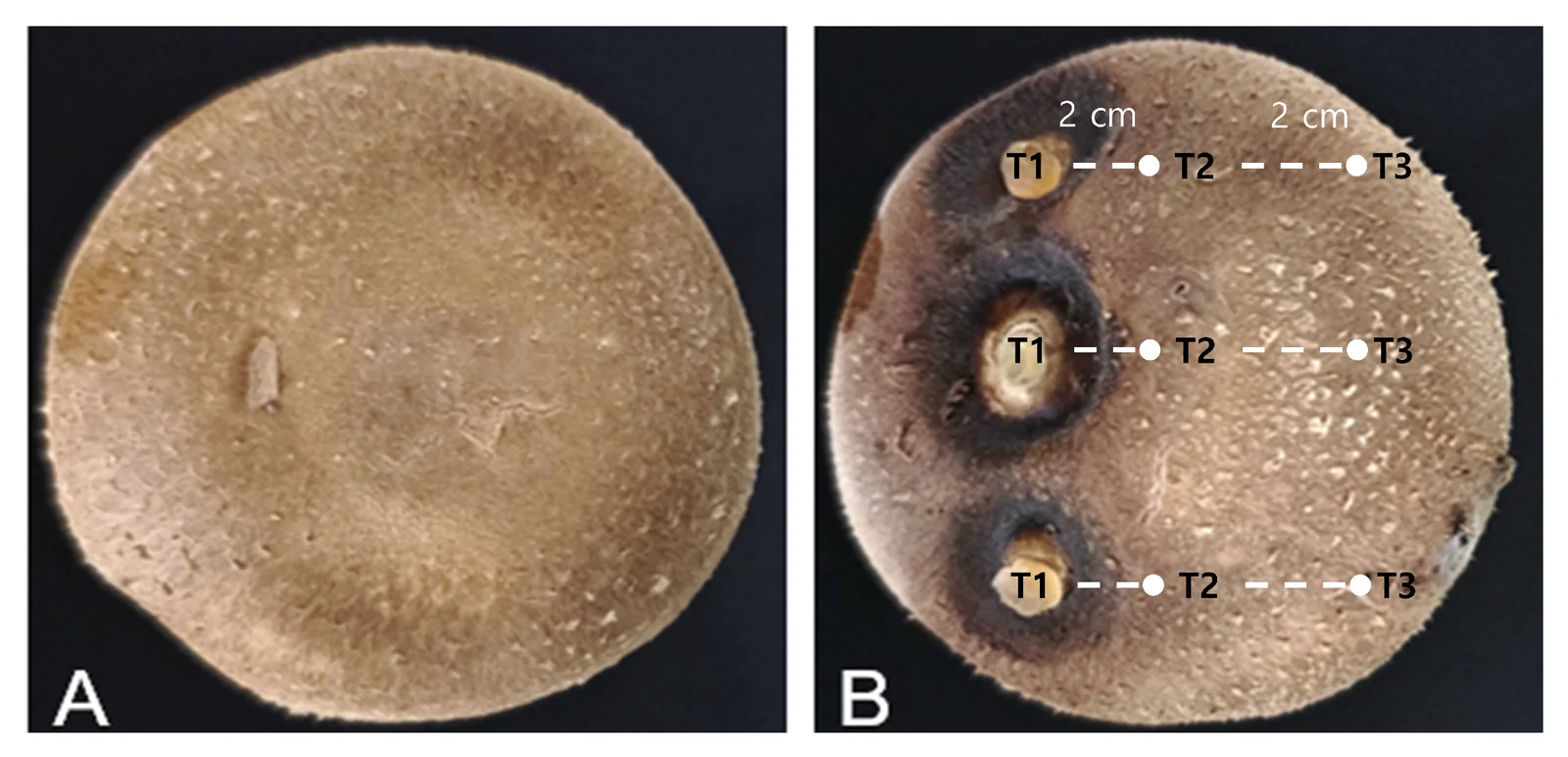
Surface symptoms on the *Lentinula edodes pileus* 3 days after *Trichoderma atroviride* inoculation. (**A**) Non-inoculated control pileus. (**B**) *T. atroviride*-inoculated pileus showing three visible lesions and the spatial sampling positions used for VOC analysis. T1 represents the three inoculation sites, whereas T2 and T3 were located 2 and 4 cm from the corresponding T1 sites, respectively, along three approximately parallel sampling transects. The dashed lines and white circles indicate the sampling transects and sampling positions, respectively.

**Figure 3:**
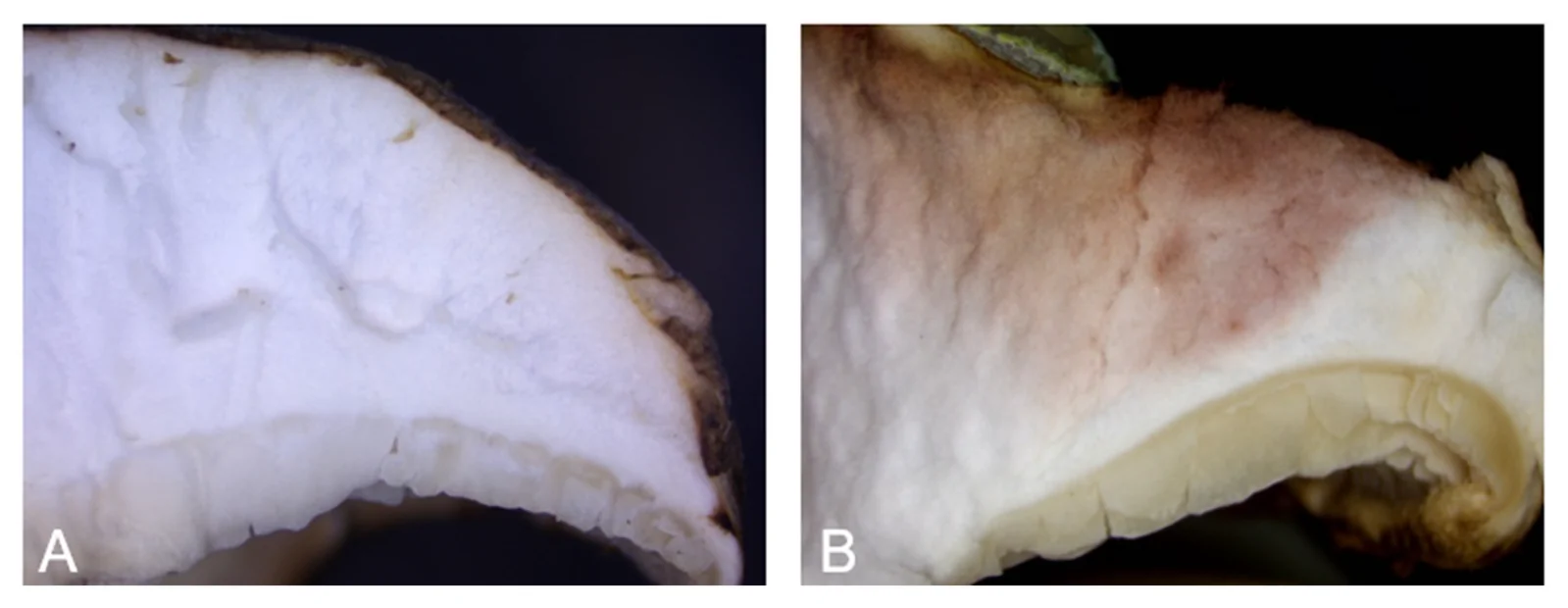
Stereomicroscopic images *of Lentinula edodes* pileus cross-sections 3 days after *Trichoderma atroviride* inoculation. (**A**) Non-inoculated control. (**B**) *T. atroviride*-inoculated pileus.

At 3 days after inoculation with *T. atroviride*, clear disease symptoms were observed on the pileus of *L. edodes* (Figure 2 and Figure 3). Observation of the inoculated pileus surface showed that no visible change was detected in the control, whereas in the inoculated pileus, a depressed lesion centered on the inoculation site and browning in the surrounding area were observed (Figure 2). In addition, stereomicroscopic examination of pileus cross-sections revealed that the control exhibited a relatively uniform white coloration in the internal tissue, whereas cross-sections inoculated with *T. atroviride* showed browning and pink discoloration in the tissue beneath and adjacent to the inoculation site (Figure 3). These results indicate that the symptoms induced by *T. atroviride* inoculation were not confined to the surface but were also manifested within the internal tissue. Following the incubation period, *T. atroviride* was re-isolated from each sampling position to verify the presence of viable fungal cells. The viable counts were (7.23 ± 0.31) × 10^3^ CFU/g at T1, (1.50 ± 0.20) × 10^3^ CFU/g at T2, and (4.67 ± 0.58) × 10^2^ CFU/g at T3. The highest viable count was observed at T1, the inoculation site, whereas the lowest count was detected at T3, the position farthest from the inoculation site. Viable colonies of *T. atroviride* were recovered from all three sampling positions, confirming that the fungus remained present in the T1, T2, and T3 tissues at 3 days after inoculation.

### 3.3. Visualization of VOC Patterns Among Sample Groups Using PCA and Heatmap Analysis

**Figure 4:**
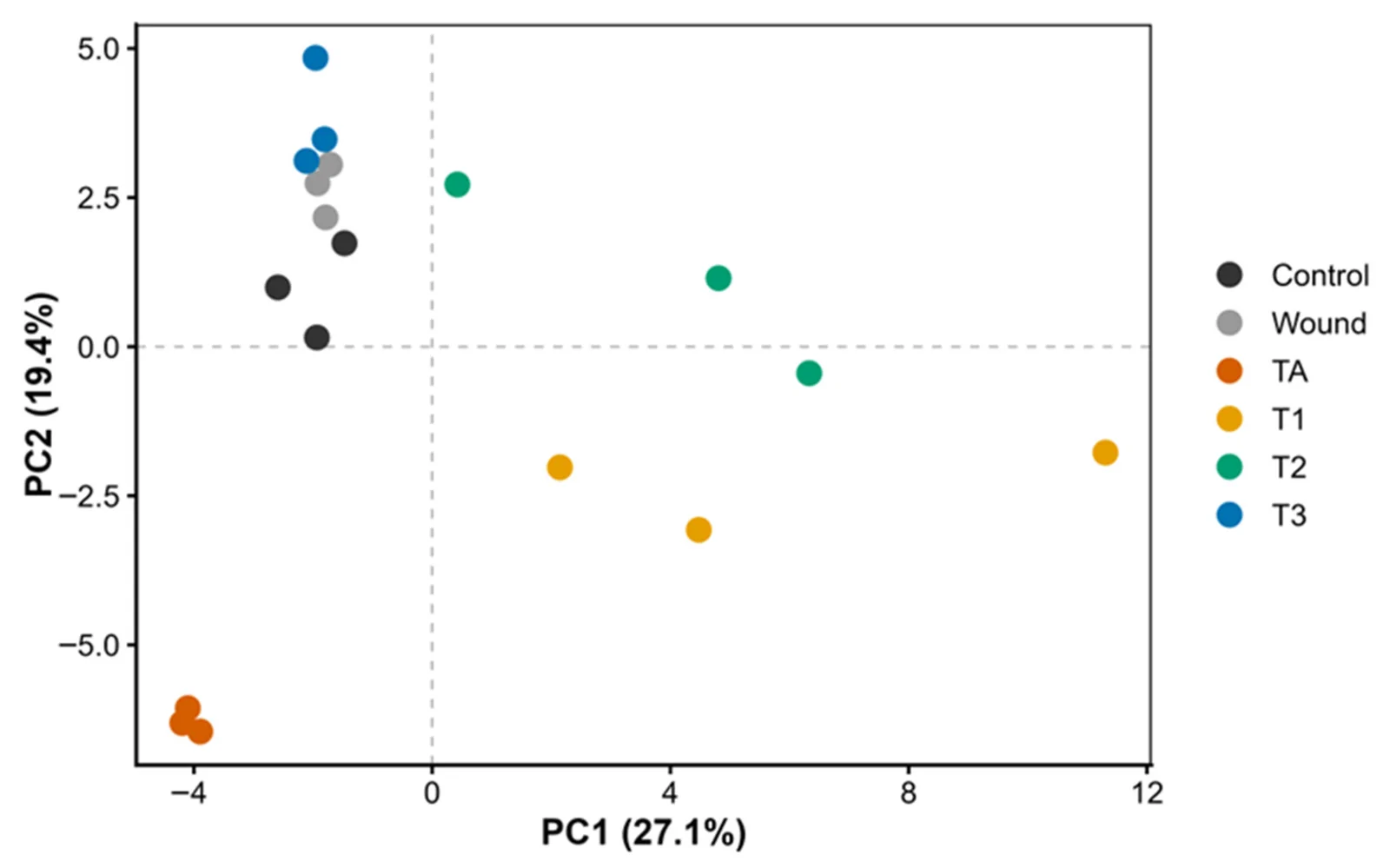
PCA of 65 background-filtered volatile organic compounds among the experimental groups. Principal component analysis (PCA) based on the relative peak area percentages of 65 background-filtered VOCs in healthy pileus tissues (Control), mechanically wounded pileus tissues (Wound), *T. atroviride* cultures (TA), and position-specific samples collected from inoculated pilei (T1, T2, and T3). PC1 and PC2 explained 27.1% and 19.4% of the total variance, respectively. Control and Wound tended to be distributed separately from TA, whereas T1 and T2 showed relatively greater within-group dispersion, and T3 formed a comparatively compact distribution. The gray dashed lines indicate zero values on the PC1 and PC2 axes.

Principal component analysis (PCA) was performed to compare VOC patterns among healthy pileus tissues (Control), the wound-only group, *T. atroviride* cultures (TA), and position-specific samples collected from inoculated pilei (T1, T2, and T3) (Figure 4). PC1 and PC2 explained 27.1% and 19.4% of the total variance, respectively. The control and wound-only samples showed distributional tendencies distinct from those of the TA samples, while the T3 samples were positioned relatively closer to the control and wound-only samples than to the TA samples. In contrast, T1 and T2 showed relatively broad dispersion among replicates.

**Figure 5:**
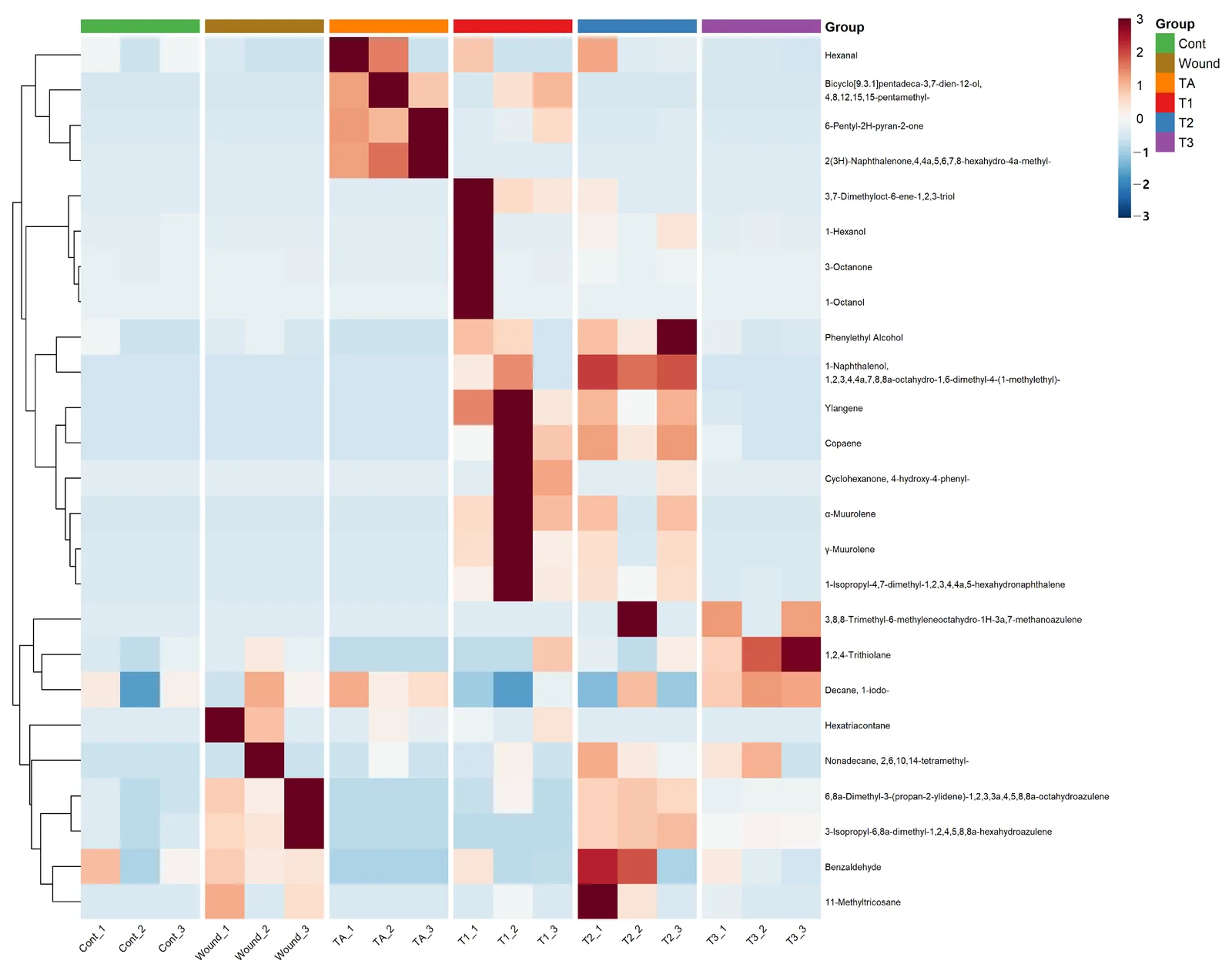
Heatmap of the 25 most variable volatile organic compounds across healthy, wounded, inoculated, and *T. atroviride* culture samples. The heatmap shows the relative patterns of the 25 VOCs with the highest variance across healthy pileus tissues (Control), mechanically wounded pileus tissues (Wound), *T. atroviride* culture samples (TA), and position-specific samples collected from inoculated pilei (T1, T2, and T3). Rows represent VOCs, and columns represent biological replicates. Values are shown as z-score-normalized relative peak area percentages. VOC rows were hierarchically clustered using Euclidean distance and complete linkage, whereas sample columns were arranged by group without clustering.

A heatmap was generated using the 25 VOCs with the highest variance across all samples among the 65 VOCs retained after background peak filtering (Figure 5). The heatmap revealed exploratory variation in relative VOC patterns among the experimental groups. The Control and Wound samples showed broadly similar patterns, although relatively high z-scores were observed for some VOCs depending on the treatment group or biological replicate. The TA samples showed a visually distinguishable pattern, with relatively high z-scores for a specific cluster of VOCs. Among the inoculated pileus samples, T1 showed relatively high heterogeneity among biological replicates, whereas T2 exhibited variable patterns depending on the VOC. In contrast, the T3 replicates showed comparatively similar patterns, and relatively high z-scores were limited to a smaller subset of VOCs. Overall, the heatmap patterns were broadly consistent with the PCA results, particularly with respect to the distinct VOC profile of TA and the greater within-group variation observed in T1 than in T3.

### 3.4. Comparison of Representative VOCs Among Control, Wound-Only, TA, and Inoculated Pileus Samples

To examine the overall differences in VOC patterns identified by the PCA and heatmap analyses in greater detail, representative VOCs showing different detection patterns among the control, wound-only, TA, and position-specific samples collected from *T. atroviride*-inoculated pilei (T1–T3) were compared (Table 2). Some VOCs were detected primarily in TA, whereas others were not detected in the control, wound-only, or TA samples but were observed in the inoculated pileus tissues, indicating different detection patterns among the sample groups. Comparison among the T1–T3 positions showed that only one compound had a raw *p*-value below 0.05; however, no compounds remained significant after FDR correction (Table S1).

In TA, 6-pentyl-2H-pyran-2-one was detected at the highest level, whereas 2(3H)-naphthalenone was consistently detected only in TA. In contrast, ylangene, γ-muurolene, α-muurolene, and 1-naphthalenol were not detected in the control, wound-only, or TA samples but were observed in the inoculated pileus samples, primarily at T1 and T2. Meanwhile, 3-isopropyl-6,8a-dimethyl-1,2,4,5,8,8a-hexahydroazulene showed its highest peak area percentage in the wound-only samples.

At T1, the inoculation site, γ-muurolene, α-muurolene, 3-octanone, and 3,7-dimethyloct-6-ene-1,2,3-triol showed their highest peak area percentages. At T2, 1-naphthalenol and phenylethyl alcohol were detected at relatively high levels. In contrast to these compounds, which generally showed higher values at T1 or T2, 1,2,4-trithiolane showed its highest value at T3, the sampling position farthest from the inoculation site.

These results indicate that the relative detection patterns of representative VOCs differed among the control, wound-only, TA, and position-specific samples collected from the inoculated pilei.

## 4. Discussion

In this study, phylogenetic analyses based on ITS and *tef1* sequences confirmed that the strain responsible for inducing disease symptoms in the *L. edodes* pileus was *T. atroviride*. *Trichoderma* spp. are well known as pathogenic competitive fungi associated with green mold disease in the cultivation of edible mushrooms, including *L. edodes*, and the present finding is consistent with previous studies reporting that these fungi damage substrates or logs and inhibit mushroom growth during cultivation [21,22]. In the *L. edodes* pileus inoculated with *T. atroviride*, surface depression and browning were observed around the inoculation site, and examination of the cross-section revealed browning and pink discoloration in the tissue beneath and adjacent to the inoculation site. These results suggest that the disease symptoms were not confined to the surface of the inoculation site but were also associated with changes at the level of internal tissue. Previous studies have likewise reported that symptoms caused by *Trichoderma* spp. may induce brown necrotic spots and lesions in mushroom fruiting bodies [4,23]. In addition, re-isolation of the inoculated strain yielded viable colonies from all T1, T2, and T3 sampling positions. Viable counts were highest at T1, the inoculation site, and lowest at T3, the position farthest from the inoculation site. These results indicate that viable fungal cells were present in the T1, T2, and T3 tissues at the time of VOC analysis.

Host–pathogen interactions may manifest heterogeneously even within the same tissue owing to differences in the local microenvironment, pathogen establishment, and the nature of the interaction itself, and thus may produce different outcomes depending on the site of contact [24,25]. In addition, the composition of VOCs produced by *Trichoderma* spp. has been reported to vary according to species, culture medium, the physiological state of the fungus, and the duration of interaction with other microorganisms [26,27,28]. The fruiting body of *L. edodes* may likewise exhibit distinct VOC profiles according to tissue region, and tissue-specific volatile variation among the pileus skin, gills, and stipe has previously been reported [11]. In the present study, following *T. atroviride* inoculation, T1, T2, and T3 exhibited different relative VOC compositions and profile patterns, whereas the wound-only control showed an overall VOC profile relatively similar to that of the healthy control. This suggests that the differences observed among T1–T3 may not be fully explained by mechanical injury alone. Given that T1 corresponded to the *T. atroviride* inoculation site, the greater dispersion observed among the T1 replicates may be associated with biological heterogeneity near the inoculation site. In contrast, T3 showed relatively lower variation among replicates and a more compact distribution than T1, and its PCA distribution showed partial similarity to those of the healthy and wound-only controls. These findings suggest that changes in the VOC profile may have been relatively limited at T3 compared with regions closer to the inoculation site. However, because this study was based on relative VOC profiling using peak area percentages, it did not measure absolute concentrations or emission rates, and included a limited number of biological replicates, the differences observed among T1–T3 should be interpreted as exploratory spatial patterns for future investigation rather than as definitive position-dependent responses. 

Heatmap analysis likewise showed that the healthy control and wound-only control exhibited broadly similar VOC patterns, whereas TA displayed a visually distinct profile. This distinction is consistent with previous reports that microbial VOC profiles may vary depending on co-culture conditions and the stage of interaction, and that pure cultures can exhibit profiles distinct from those observed under interaction conditions [29,30]. Among the inoculated samples, T1 replicates showed marked heterogeneity, whereas T3 replicates exhibited relatively similar patterns. This tendency was consistent with the PCA results and suggested exploratory differences in relative VOC patterns among the T1, T2, and T3 sampling positions.

Comparison of the detection patterns of representative VOCs revealed distinct VOC profiles between the TA monoculture and the inoculated pileus tissues, and different detection patterns were also observed among sampling positions within the inoculated pileus. In TA, 2(3H)-naphthalenone was consistently detected only in this group, whereas 6-pentyl-2H-pyran-2-one showed the highest relative peak area percentage. However, 6-pentyl-2H-pyran-2-one was also detected in T1 and in some T2 replicates and was therefore not restricted to the TA monoculture. 6-Pentyl-2H-pyran-2-one is known as a representative volatile compound of T. atroviride, and its reported biological functions include the modulation of plant responses and antifungal activity [31,32]. In addition, 2(3H)-naphthalenone has previously been detected during the culture of *Trichoderma* spp. [33]. Therefore, the detection patterns of these compounds may be interpreted as partially reflecting culture-associated characteristics related to the growth of *T. atroviride*.

In contrast, ylangene, γ-muurolene, α-muurolene, 1-naphthalenol, and 3,7-dimethyloct-6-ene-1,2,3-triol were not detected in the healthy control, wound-only control, or TA samples but were detected in the inoculated pileus tissues. Their absence from the wound-only control suggests that the detection patterns observed in the inoculated pileus cannot be fully explained by mechanical injury alone and may be associated with host–fungus interactions following inoculation. Specifically, ylangene was detected at T1, T2, and T3, whereas γ-muurolene and α-muurolene were detected mainly at T1 and T2, and 1-naphthalenol was detected mainly at T2 and at lower levels at T1. These patterns suggest possible spatial variation within the inoculated pileus, with some compounds detected near the inoculation site and others detected across a broader range of sampling positions. Pathogen infection can alter the volatile composition of mushrooms [10], and changes in sesquiterpene VOCs, including α-ylangene, have also been reported in plant–fungus interactions [34,35].

However, not all compounds appeared to be specifically associated with inoculation. 3-Isopropyl-6,8a-dimethyl-1,2,4,5,8,8a-hexahydroazulene showed the highest relative peak area percentage in the wound-only control. Similarly, 3-octanone, phenylethyl alcohol, and 1,2,4-trithiolane were also detected in the healthy control and wound-only control groups. Although 3-octanone showed the highest relative peak area percentage at T1, it was also detected at lower levels in the healthy control, wound-only control, T2, and T3 samples. Because this compound has previously been reported as a ketone-type volatile in mushrooms, including *L. edodes* [36], its occurrence may reflect the inherent volatile background of the mushroom rather than a response specific to *T. atroviride* inoculation.

Likewise, 1,2,4-trithiolane showed the highest relative peak area percentage at T3 but was also detected in the healthy control, wound-only control, T1, and T2 samples. Because 1,2,4-trithiolane has been reported as a representative sulfur-containing volatile of shiitake [37,38], its relatively high value at T3 should be interpreted in the context of the inherent sulfur-volatile profile of *L. edodes* rather than as evidence of a *T. atroviride*-specific VOC response. Therefore, variation in these compounds may be associated with mechanical injury, inherent VOC variation in the fruiting body, or relative compositional differences among treatments rather than with a response specific to *T. atroviride* inoculation. Furthermore, because no compounds remained significant after correction for multiple comparisons, the detection patterns of individual VOCs should be interpreted as exploratory patterns requiring further validation rather than as definitive position-specific responses.

This study was conducted as an exploratory investigation to determine whether the VOC composition and profiles of *L. edodes* pileus tissues may vary with distance from the inoculation site following *T. atroviride* inoculation. Because VOC profiles were analyzed only at a single time point, 3 days after inoculation, the present study cannot distinguish whether the observed changes were associated with early pathogen establishment, ongoing host responses, or later tissue deterioration. Time-course analyses combining pathogen abundance and VOC profiling at multiple stages of infection will therefore be necessary to characterize the temporal dynamics of these responses. However, because the present analysis was based on relative VOC profiling using peak area percentages, the current data do not allow quantification of the absolute concentrations or emission rates of the detected compounds, nor do they permit clear determination of the biological origin and functional role of each VOC. Therefore, future studies should quantitatively analyze the compounds showing different detection patterns among T1, T2, and T3 using internal and authentic standards. The reproducibility of the spatial patterns observed in this study should also be evaluated through independent validation experiments with a larger number of biological replicates. Furthermore, parallel analysis of changes in related metabolic pathways or response-associated gene expression could provide a more detailed understanding of the relationship between potential spatial variation in VOC profiles following *T. atroviride* inoculation and the underlying molecular responses.

## 5. Conclusions

In this study, phylogenetic analyses based on ITS and *tef1* sequences supported the identification of the strain associated with disease symptom development in the *L. edodes* pileus as *Trichoderma atroviride*. Inoculation of *T. atroviride* onto the *L. edodes* pileus resulted in surface depression, browning, and discoloration of the cut surface, indicating that the symptoms extended into the internal tissues of the fruiting body. PCA and heatmap analyses showed differences in relative VOC profile patterns among the healthy control, wound-only control, *T. atroviride* culture (TA), and position-specific samples collected after inoculation (T1–T3). The wound-only control exhibited an overall VOC profile broadly similar to that of the healthy control, whereas the inoculated samples showed exploratory spatial differences in relative VOC composition. Analysis of representative VOCs further distinguished culture-associated VOCs of TA from compounds showing different detection patterns within the inoculated pileus. Taken together, the present study provides a useful basis for further quantitative investigation of inoculation-associated VOC changes and potential spatial variation within the *L. edodes* pileus.

## Figures and Tables

**Table 1:** SPME-GC-MS analytical conditions for VOCs profiling.

Section	Parameter	Condition
SPME	Fiber	65 µm DVB/PDMS
	Conditioning temperature	200 °C
	Pre-conditioning time	10 min
	Incubation temperature	60 °C
	Incubation time	30 min
	Agitation speed	250 rpm
	Extraction time	10 min
	Desorption time	1 min
	Post-conditioning time	3 min
GC	Column	HP-5ms UI capillary column (30 m × 0.25 mm, 0.25 µm)
	Carrier gas	Helium
	Injection mode	Splitless
	Injection temperature	250 °C
	Column flow	2.0 mL/min
	Oven program	40 °C for 3 min, ramped at 4 °C/min to 250 °C, held for 5 min
MS	Ionization	EI
	Electron energy	70 eV
	Ion source temperature	230 °C
	Interface temperature	150 °C
	Scan range	*m*/*z* 45–500

**Table 2:** Detection patterns of selected tentatively annotated volatile organic compounds (VOCs) in *T. atroviride* culture, healthy *L. edodes* pileus, wound-only controls, and inoculated pileus samples.

Compound	RT (min)	Chemical Class	Detection Pattern
6-Pentyl-2H-pyran-2-one	27.18	Lactone/Pyrone	Not detected in Cont, Wound, or T3; highest in TA; also detected in T1 and in one T2 replicate.
2(3H)-Naphthalenone	29.07	Ketone derivative	Detected consistently only in TA; not detected in Cont, Wound, or inoculated pileus samples.
γ-Muurolene	27.65	Sesquiterpene hydrocarbon	Not detected in Cont, Wound, TA, or T3; detected mainly in T1 and at lower levels in T2.
α-Muurolene	28.38	Sesquiterpene hydrocarbon	Not detected in Cont, Wound, TA, or T3; detected mainly in T1 and at lower levels in T2.
Ylangene	24.25	Sesquiterpene hydrocarbon	Not detected in Cont, Wound, or TA; detected in T1 and T2, with trace-level detection in T3.
3-Octanone	10.53	Ketone	Detected at low levels in Cont, Wound, T2, and T3, absent in TA, and highest in T1, with substantial variation among T1 replicates.
3,7-Dimethyloct-6-ene-1,2,3-triol	30.51	Polyol/alcohol	Not detected in Cont, Wound, TA, or T3; the highest mean peak-area percentage was observed in T1, with detection in one T2 replicate.
1-Naphthalenol	32.71	Bicyclic aromatic alcohol	Not detected in Cont, Wound, TA, or T3; detected mainly in T2 and at lower levels in T1.
Phenylethyl Alcohol	15.17	Aromatic alcohol	Detected in Cont, Wound, and all inoculated pileus groups, but not in TA; the highest mean peak-area percentage was observed in T2.
3-Isopropyl-6,8a-dimethyl-1,2,4,5,8,8a-hexahydroazulene	29.20	Sesquiterpene-related hydrocarbon	Detected in Cont, Wound, T2, and T3, but not in TA or T1; the highest mean peak-area percentage was observed in Wound, followed by T2 and T3.
1,2,4-Trithiolane	14.17	Sulfur-containing heterocycle	Detected in Cont, Wound, T1, T2, and T3, but not in TA; the highest mean peak-area percentage was observed in T3.

The detection patterns shown in this table represent qualitative observations and do not indicate statistically significant positional differences. Detailed statistical results are provided in Table S1.

## Data Availability

The original contributions presented in this study are included in the article/Supplementary Material. Further inquiries can be directed to the corresponding authors.

## Supplementary Materials

The following supporting information can be downloaded at: https://www.mdpi.com/article/10.3390/jof12080583/s1, Table S1. Exploratory Friedman test results for representative VOCs among T1, T2, and T3 samples of inoculated Lentinula edodes pileus. Values are presented as mean ± SD (*n* = 3). Raw *p*-values and Benjamini–Hochberg FDR-adjusted p-values are shown. Compounds with all-zero values across T1–T3 were considered not testable.
