# Supplementary material for: Volatile Organic Compound Profile Changes in Lentinula edodes Pileus Following Trichoderma atroviride Inoculation
Source: J Fungi (Basel). 2026 Aug 7;12(8):583. doi: 10.3390/jof12080583 (PMC13514159; doi:10.3390/jof12080583)
Supplement: Supplementary file 1 [file jof-12-00583-s001.zip › jof-4430995-supplementary.pdf]

Table S1. Exploratory Friedman test results for representative VOCs among T1, T2, and T3 samples of inoculated *Lentinula edodes* pileus. Values are presented as mean ± SD (n = 3). Raw p-values and Benjamini–Hochberg FDR-adjusted p-values are shown. Compounds with all-zero values across T1–T3 were considered not testable.

Compound	T1	T2	T3	Friedman	P_value	FDR_BH	Note
**2H-Pyran-2-one, 6-pentyl-**	5.283± 6.399	0.257± 0.445	0.000±0.000	5.6	0.0608	0.152	
**2(3H)-Naphthalenone**	0.000± 0.000	0.000± 0.000	0.000±0.000	NA	NA	NA	All zero in T1-T3; not testable
**γ-Muurolene**	2.473± 2.527	0.827± 0.716	0.000±0.000	3.818	0.148	0.185	
**α-Muurolene**	1.363± 0.975	0.587± 0.508	0.000±0.000	3.818	0.148	0.185	
**Ylangene**	1.853± 1.137	1.070± 0.538	0.050±0.062	4.667	0.097	0.162	
**3-Octanone**	1.043± 1.712	0.110± 0.044	0.063±0.006	1.273	0.529	0.529	
**3,7-Dimethyloct-6-ene-1,2,3-triol**	0.353± 0.353	0.040± 0.069	0.000±0.000	5.6	0.0608	0.152	
**1-Naphthalenol**	0.253± 0.272	0.663± 0.060	0.000±0.000	5.636	0.0597	0.152	
**Phenylethyl Alcohol**	0.473± 0.382	1.083± 0.841	0.117± 0.064	2	0.368	0.409	
**Azulene-related sesquiterpene**	0.000± 0.000	0.423± 0.035	0.223± 0.021	6	0.0498	0.152	
**1,2,4-Trithiolane**	0.130±0.225	0.117±0.120	0.643±0.280	4.909	0.0859	0.162	
